# Complete Genome Sequence of Agrobacterium tumefaciens Myophage Milano

**DOI:** 10.1128/MRA.00587-19

**Published:** 2019-06-20

**Authors:** Toni Nittolo, Aravind Ravindran, Carlos F. Gonzalez, Jolene Ramsey

**Affiliations:** a Center for Phage Technology, Texas A&M University, College Station, Texas, USA; b Department of Plant Pathology and Microbiology, Texas A&M University, College Station, Texas, USA; DOE Joint Genome Institute

## Abstract

Agrobacterium tumefaciens C58 is a tumor-causing pathogen targeting plants and is ubiquitously found in soil. Here, the complete genome sequence of Milano, a myophage infecting A. tumefaciens C58, is presented. Milano encodes 127 proteins, of which 45 can be assigned a predicted function, and it is most similar to the flagellotropic *Agrobacterium* phage 7-7-1.

## ANNOUNCEMENT

Agrobacterium tumefaciens C58 is a Gram-negative rod-shaped bacterium ubiquitously found in soil (1). As a plant pathogen, A. tumefaciens C58 contains plasmid TiC58 that transfers transfer DNA (T-DNA) to 90 families of dicotyledonous plants, inevitably resulting in crown gall tumors. Bacteriophages may be useful in manipulating this characteristic for engineering *Agrobacterium* strains. Here, we present the genome sequence of myophage Milano.

Milano was isolated from filtered rice stem extracts in Beaumont, TX, on A. tumefaciens C58 grown aerobically at 28°C in nutrient broth yeast (NBY) medium without glucose (2) by the soft agar overlay method (3). The high-titer lysate generated via the soft agar overlay method was used for extracting genomic DNA with the phenol-chloroform method, as in reference 4, and then the phage genomic DNA libraries were prepared using a NEBNext Ultra II DNA library prep kit and sequenced on an Illumina MiSeq instrument at the Genome Sequencing and Analysis Facility at the University of Texas at Austin (5). The 820,950 250-bp paired-end sequence reads were quality controlled with FastQC (http://www.bioinformatics.babraham.ac.uk/projects/fastqc/) and trimmed with the FASTX-Toolkit 0.0.14 (hannonlab.cshl.edu/fastx_toolkit/). Complete assembly into a single contig using SPAdes v.3.5.0, with default parameters, was confirmed via PCR of the genome ends (forward primer, 5′-GTCCTGAATCCATTTCGTATGC-3′; reverse primer, 5′-CTCCGTTCTTCAGCTACTATG-3′), coupled with Sanger sequencing (5, 6). Genes were called and annotated using GLIMMER 3.0 and MetaGeneAnnotator 1.0 in the Web Apollo instance hosted by the Center for Phage Technology (https://cpt.tamu.edu/galaxy-pub), and all analyses were performed in their Galaxy instance (7–10). Potential tRNA genes were inspected using ARAGORN 2.36 (11). Gene functions were predicted using domains from InterProScan v.5.22, LipoP, and TMHMM, as well as BLASTp comparisons to the NCBI nonredundant (nr) and UniProtKB Swiss-Prot/TrEMBL databases (12–16). HHpred results were used as confirmatory evidence, in addition to the presence of domains or alignments (17). Rho-independent termination sites were detected using TransTerm (http://transterm.cbcb.umd.edu/). Milano’s morphology was determined by negative-stain transmission electron microscopy at the Texas A&M Microscopy and Imaging Center with 2% (wt/vol) uranyl acetate (18).

Milano is a myophage with a 68,451-bp genome, 93.1% coding density, and a G+C content of 52.5%, which is lower than the G+C content of 58% of the host, A. tumefaciens C58 (1). Our analysis revealed 127 coding sequences, of which 45 have a function predicted by using BLASTp or InterProScan, but no tRNAs. Based on PhageTerm prediction, Milano uses headful DNA packaging (19). While Milano contains mostly hypothetical proteins, there is one closely related phage in the NCBI nr database, namely, *Agrobacterium* myophage 7-7-1 (GenBank accession number JQ312117) (20). By comparison with progressiveMauve, Milano has 61.68% nucleotide identity and shares 94 proteins with *Agrobacterium* phage 7-7-1, a flagellotropic phage (21).

The Milano genome is organized in a modular fashion, with predicted structural, replication, and lysis proteins grouped together. The predicted tape measure protein (GenBank accession number QBQ72047) is preceded by the likely tail assembly chaperones (QBQ72045) with a putative frameshifted protein product (QBQ72046), analogous to the well-studied lambda G/GT chaperone system (22). The predicted endolysin (QBQ72055), i-spanin (QBQ72056), and embedded o-spanin (QCQ78506) are encoded consecutively; however, the holin was not identified. Additionally, a putative nucleoid occlusion-like protein (QBQ72073) with a ParB domain (InterProScan IPR003115) and many BLASTp hits to Noc proteins and two potential ribosome modulation factor domain superfamily proteins (QBQ72082 and QBQ72098) were found.

### Data availability.

The genome sequence and associated data for phage Milano were deposited under GenBank accession number MK637516, BioProject accession number PRJNA222858, SRA accession number SRR8869236, and BioSample accession number SAMN11360273.
